# Nanoparticle-Mediated Delivery of Emodin via Colonic Irrigation Attenuates Renal Injury in 5/6 Nephrectomized Rats

**DOI:** 10.3389/fphar.2020.606227

**Published:** 2021-01-21

**Authors:** Zhaoyu Lu, Chunlan Ji, Xuewen Luo, Yong Lan, Lijuan Han, Yang Chen, Xusheng Liu, Qinzhan Lin, Fuhua Lu, Xiuqing Wu, Rui Guo, Chuan Zou

**Affiliations:** ^1^ The Second Clinical Medical College, Guangzhou University of Chinese Medicine, Guangzhou, China; ^2^ Department of Nephrology, Guangdong Provincial Hospital of Chinese Medicine, Guangzhou, China; ^3^ Department of Nephrology, The Affiliated Hospital of Jiangxi University of Chinese Medicine, Nanchang, China; ^4^ Department of Biomedical Engineering, Jinan University, Guangzhou, China; ^5^ Department of Bioinformatics, Guangdong Provincial Hospital of Chinese Medicine, Guangzhou, China

**Keywords:** nanoparticles, chronic kidney disease, emodin, gut microbiota, irrigation

## Abstract

Our previous study showed that emodin enema modulates gut microbiota and delays CKD progression. However, the poor solubility, limited colonic irrigation retention time, and inadequate colon adhesion of emodin hinder its clinical application. Based on the deficiencies of emodin, we prepared monomethoxy-poly (ethylene glycol)-poly (lactic acid)-chitosan-2-mercaptobenzimidazole nanoparticles with incorporated emodin (emodin-NP) and studied their efficacy in delaying CKD progression. 5/6 nephrectomized Male Sprague Dawley rats were administered via colonic irrigation with emodin-NP every two days for eight weeks. We found that treatment with emodin-NP improved the kidney function of the rats and limited the expansion of tubulointerstitial fibrosis. Treatment with emodin-NP once every two days is comparable to emodin treatment once a day. Furthermore, emodin-NP via colonic irrigation remarkably reduced IL-1β, IL-6, and LPS levels in serum, improved intestinal barrier functions, and downregulated the key proteins (TLR4, MyD88, and NF-κB) expression in intestinal TLR4 signaling pathway. 16S rDNA analyses showed that emodin-NP can regulate microbiota disturbance in CKD. Taken together, these results suggest that emodin-NP alleviates kidney dysfunction and tubulointerstitial fibrosis by mediation through the modification of gut microbiota disorders. Emodin-NP may be a new method to treat CKD.

## Introduction

Chronic kidney disease (CKD) seriously affect human health worldwide. It would be beneficial for patients, the economy, and health care systems to delaying the progression of CKD into end-stage renal disease (Ojo, 2014; Glassock et al., 2017). Many studies have indicated that CKD is associated with intestinal flora disorders and intestinal barrier injury. Damage to the intestinal barrier in CKD causes enterogenic uremia toxins to enter into circulation, inducing systemic inflammation and kidney injury (Ramezani and Raj, 2014; Ramezani et al., 2016).

In Chinese medicine (CM) theory, the elimination of uremic toxins is key to the treatment of CKD (Guo and Rao, 2018). Rhubarb has been widely employed since ancient times to reduce uremic toxins (Wang et al., 2012; Zhong et al., 2013), and colonic irrigation with rhubarb-based decoctions has been used by CM practitioners for half a century as a CKD treatment. Emodin (1,3,8-trihydroxy-6-methylanthraquinone) is the most important component of rhubarb (Dong et al., 2016). Our foregone study showed that irrigation with emodin modulates gut microbiota and uremic toxin levels through the gut-kidney axis to treat CKD (Zeng et al., 2016). However, the poor solubility, limited colonic irrigation time, and inadequate colon adhesion of emodin hinder its effects on the intestinal microecology. Currently, there is a lack of studies to improve these problems.

In this study, we prepared a colon-targeting drug delivery system consisting of polymer nanoparticles. The system is a new core-shell bi-block copolymer conjugate comprising monomethoxy-poly (ethylene glycol)-poly (lactic acid)-chitosan-2-mercaptobenzimidazole (mPEG-PLA-CS-MBI) nanoparticles. PEG, CS, PLA, and MBI have been approved for medical applications by the Food and Drug Administration (Muxika et al., 2017). Next, we combined emodin with the nanoparticles to prepare an emodin-nanoparticle system (emodin-NP). We hypothesized that the colon-targeted emodin-NP would have a higher stability and better colon adhesion and persistence effects than emodin alone. This could improve the emodin colonic irrigation treatment in terms of regulating gut microbiota disorders, improving intestinal mucosal barrier functions, and thus having a better therapeutic effect on CKD.

## Materials and Methods

### Materials

Anti-TLR4 was purchased from Santa Cruz (Dallas, TX, United States), anti-NF-κB and *β*-actin were purchased from Cell Signaling Technologies (Darmstadt, Germany), anti-MyD88 and anti-collagen Ⅰ were obtained from Abcam (Cambridge, MA, United States). HRP-conjugated anti-mouse IgG and rabbit IgG were purchased from Cell Signaling Technologies. The enhanced chemiluminescence reagent and Image Lab System were obtained from Bio-Rad (Hercules, CA, United States), and the BCA protein detection kit was procured from Thermo Fisher Scientific (Waltham, MA, United States). IL-1β and IL-6 assay kits were purchased from Abcam, TNF-α, IFN-γ assay kits were obtained from Raybiotech (Georgia, United States), and the LPS assay kit was obtained from Aviva Systems Biology (CA, United States). The hematoxylin and eosin (HE) staining kit was obtained from BOSTER (Wuhan, China), and the Masson staining kit was purchased from Nanjing Jiancheng Bioengineering Institute (Jiangsu, China).

Emodin was purchased from Shanghai Winherb Medical Technology Company (Shanghai, China). PLA was purchased from Jinan Daigang Biomaterial Company, Shandong, China, and mPEG was purchased from Xian Ruixi Biomaterial Company, Shanxi, China.

### Synthesis of mPEG-PLA-OH

mPEG-PLA-OH was obtained by dissolving 14.4 g L-lactide, 7.6 g mPEG together with 0.2 g stannous octanoic acid in 20 ml dichloromethane and reacting at 130°C for 18 h. The product was then precipitated in ice ether 3 times and vacuum dried at 40°C for 3 days.

### Synthesis of mPEG-PLA-CS

10 g mPEG-PLA-OH, 2 g succinic anhydride, and 1.2 g 4-dimethylaminopyridine were dissolved in 100 ml chloroform, then triethylamine was added uniformly. The reaction was conducted at room temperature (RT) for 3 days, ether was precipitated three times. mPEG-PLA-COOH was obtained after filtration and vacuum drying at 40°C for 3 days. Then, PEG-PLA-COOH was dissolved in methylene chloride, reacted with *N*-(3-dimethylaminopropyl)-*N*′-ethylcarbodiimide hydrochloride and *N*-hydroxysuccinimide at room temperature for 24 h, then solubilized in DMSO after steaming. Subsequently, the product was added to a chitosan/DMSO solvent mixture, reacted for 24 h, subjected to dialysis for 3 days, and freeze-dried to obtain the mPEG-PLA-CS product.

### Synthesis of mPEG-PLA-CS-MBI

0.5 g mPEG-PLA-CS was dissolved in 140 ml ultrapure water, 0.3 g sodium iodate (NaIO_4_) solution was added and incubated at RT for 2 h. After that, 300 μL ethylene glycol was added, and the mixture was reacted at RT for 2 h mPEG-PLA-CS-CHO was obtained after dialysis for 3 days, freeze-dried, and preserved at 4°C. 0.5 g 5-Amino-2-mercaptobenzimidazole together with 0.2 g mPEG-PLA-CS-CHO were mixed evenly in DMSO/H_2_O (1:1) and incubated at RT for 2 h, 0.2 g NaCNBH_3_ was added, the mixture was reacted at RT for 2 days. After dialysis for 3 days, the final product (mPEG-PLA-CS-MBI) was collected.

### Preparation of Emodin-NP

Briefly, a mixture of mPEG-PLA-CS-MBI and emodin was dissolved in ethyl alcohol, the mixed solution was evaporated by rotary vacuum. The resulting film was freeze-dried overnight at 40°C, rehydrated in phosphate buffer, then bathed (60°C) for 30 min. The unencapsulated domain crystals were filtered and removed using a 0.22 μm filter membrane to obtain the emodin-NP-loaded mixed polymer nanoparticles (emodin-NP).

### Measurement of mPEG-PLA-CS-MBI

The ^1^H NMR spectra were recorded using a 400 MHz superconducting Fourier transform nuclear magnetic resonance spectrometer (Bruker, Billerica, MA) with deuterium oxide (D_2_O) as the solvent. The infrared spectrum was obtained using an infrared spectrometer (FTIR-650, Tianjin, China). The microspheres were analyzed in the wavenumber range of 500–4000 cm^−1^ and were crushed into KBr pellets under 600 kg/cm^2^ of hydraulic pressure. The zeta potential and mean particle size distribution were measured using a Zetasizer Nano ZSP system (NanoZS90) using dynamic light scattering (DLS). The scattering angle was fixed at 90°, and the samples were measured at a constant temperature of 25°C. The data presented are the mean values of three measurements.

### Determination of Entrapment Efficiency and *in vitro* Drug Release

To determine the content of emodin in emodin-NP, the drug-loaded copolymer was dissolved in an aqueous ethanol solution, and the drug encapsulation rate and drug loading rate were calculated using the standard curve method. The *in vitro* release of emodin-NP was studied in pH 7.4 PBS. After weighing and lyophilization, emodin-NP was suspended in 2.0 ml of releasing medium and loaded into dialysis bags (MWCO 8000 Da). At the beginning of the release test, the dialysis bag sealed at the end was placed in 50.0 ml of release medium at 37°C and continuously vibrated at 100 rpm. At a predetermined time, 2.0 ml of external release medium was removed, and the same volume of fresh release medium was added to the dialysis system. The amount of emodin released was determined by UV spectrophotometry using the standard curve method.

### Animal and Experimental Design

Male Sprague Dawley rats weighing approximately 200 g were purchased from the Guangdong Experimental Animal Center (Guangzhou, China). The rats were housed in our animal facility under pathogen-free conditions and fed a standard laboratory diet with free access to water. The temperature was maintained at 18–22°C with a 12 h light/dark cycle. This study was approved by the institutional ethics review board of the Second Affiliated Hospital of Guangzhou University of Chinese Medicine.

The animals were randomly assigned to a sham-operated control group (normal) and CKD model groups. The CKD model was induced by 5/6 nephrectomy (5/6 Nx). Briefly, the rats were anesthetized by intraperitoneal injection of pentobarbital sodium (30 mg/kg), and the upper and lower two-thirds of the left kidney were removed. The right kidney nephrectomy was performed 7 days later. Rats in the sham group were only stripped of the fat sac without nephrectomy.

Eight weeks after the 5/6 nephrectomy, the rats in the CKD model group were randomly assigned to four groups: the model group, low-dose emodin-NP (1.15 mg/kg emodin) colonic irrigation group, high-dose emodin-NP (4.6 mg/kg emodin) colonic irrigation group, and emodin (4.6 mg/kg) colonic irrigation group. For the animals in the emodin-NP groups, 5 ml of the drug was administered via colonic irrigation using a lavage needle (8 cm) through the anus once every two days, while those in the emodin group were given emodin via colonic irrigation every day. Eight weeks after colonic irrigation, fecal samples were collected, all rats were sacrificed, and blood samples were collected. The kidneys were removed and preserved for further analysis.

### Biochemical Detection, Blood Pressure and Histopathological Studies

Serum creatinine was detected using a biochemical analyzer. Blood pressure is measured by blood pressure detector. The kidney and colon tissues were dehydrated and embedded to prepare paraffin specimens. The paraffin specimens were sliced and stained with HE, and the histological images were observed under a microscope.

### Systematic and Renal Inflammation Measurement

The levels of IL-6 (Abcam, Cambridge, United Kingdom), IL1β (Abcam, Cambridge, United Kingdom), TNF-α (Raybiotech, Georgia, United States), IFN-γ (Raybiotech, Georgia, United States) and lipopolysaccharide (LPS, Aviva, California, United States) in the serum and renal tissue were detected by an enzyme-linked immunosorbent assay (ELISA) in accordance with the manufacturer’s instructions**.**

### Western Blot Analysis

The colon and kidney tissues were lysed in RIPA buffer containing protease and phosphatase inhibitor cocktail (Thermo Fisher Scientific, Massachusetts, United States). The protein concentration was determined using a BCA detection kit (Thermo Fisher Scientific). The samples (50 μg) were separated using 10% SDS–PAGE and then transferred to polyvinylidene difluoride membranes (EMD Millipore, Burlington, VT, United States). The membranes were blocked with 5% nonfat milk for 1 h, then incubated with primary antibodies against TLR4 (1:500, Santa Cruz Biotechnology, California, United States), MyD88 (1:1000, Abcam, Cambridge, United Kingdom), NF-κB (1:1000, Cell Signaling Technologies, Boston, United States), CollagenⅠ(1:1000, Abcam, Cambridge, United Kingdom), or *β*-actin (1:5000, Sigma-Aldrich, Missouri, United States) overnight at 4°C. The membranes were washed three times and then incubated with an HRP-linked anti-rabbit or anti-mouse IgG (1:3000, Cell Signaling Technologies, Boston, United States) at RT. The membranes were then washed, and their immunoreactivity was measured using an enhanced chemiluminescence reagent (Bio-RAD, Bio-Rad Universal Hood II, California, United States). The signals were captured and analyzed using the Image Lab System (Bio-RAD 5.2.1).

### Changes in Gut Microbiota After Emodin-NP Colonic Irrigation Treatment

The DNA samples of gut microbiota were quantified using a NucleoSpin Soil Kit (Macherey Nagel, Biocompare, Germany) and then transferred to a PacBio sequel system for V4 region gene sequencing of the 16S rDNA gene with Hiseq 2500 (Illumina, California, United States). The PCR primers used for 16S rDNA amplicon libraries were 27F and 1492R.

PacBio circular consensus sequencing (CSS) was obtained and filtered using the CSS protocol with a predicted accuracy of >99%, corresponding to Q20. Dereplication, clustering, and chimera detection were performed using a set of USEARCH (version 11.0.667) tools (fastx_uniques, cluster_otus, and uchime2_ref using SILVA Gold as the reference database). Taxonomic ranks were predicted by nbc_tax, an implementation of the RDP naive Bayesian classifier algorithm, using rdp_16s_v16 as a reference database with a confidence value of 0.6. All statistical analyses were performed using version 3.6.0 R. Vegetarian packages were used to calculate the alpha diversity (Shannon index) and principal coordinate analysis (PCoA) based on Bray–Curtis dissimilarity matrices at the genus level of 16S rDNA gene sequencing. The comparison of alpha diversity between groups was performed using the Kruskal Wallis test. Dissimilarity matrices were calculated using permutational multivariate analysis of variance to assess the effects of the groups. The significant differences between the groups and their effect sizes were identified by linear discriminant analysis of effect size (LEfSe).

### Statistical Methods

SPSS statistical software (version 19.0, IBM, Armonk, NY, United States) was used to analyze and evaluate the measurement data. An independent samples *t*-test was used to compare the normal distribution data and mean square error between the two groups. Single-factor analysis of variance for multiple sets of data. The nonparametric the Mann–Whitney *U* test was used to compare two groups between heterogeneous variance of non-normal distribution data. For nonparametric rank and rank of data between tests used to compare groups, *p* < 0.05 was considered statistically significant.

## Results

### Characterization of Emodin-NP

**FIGURE 1:**
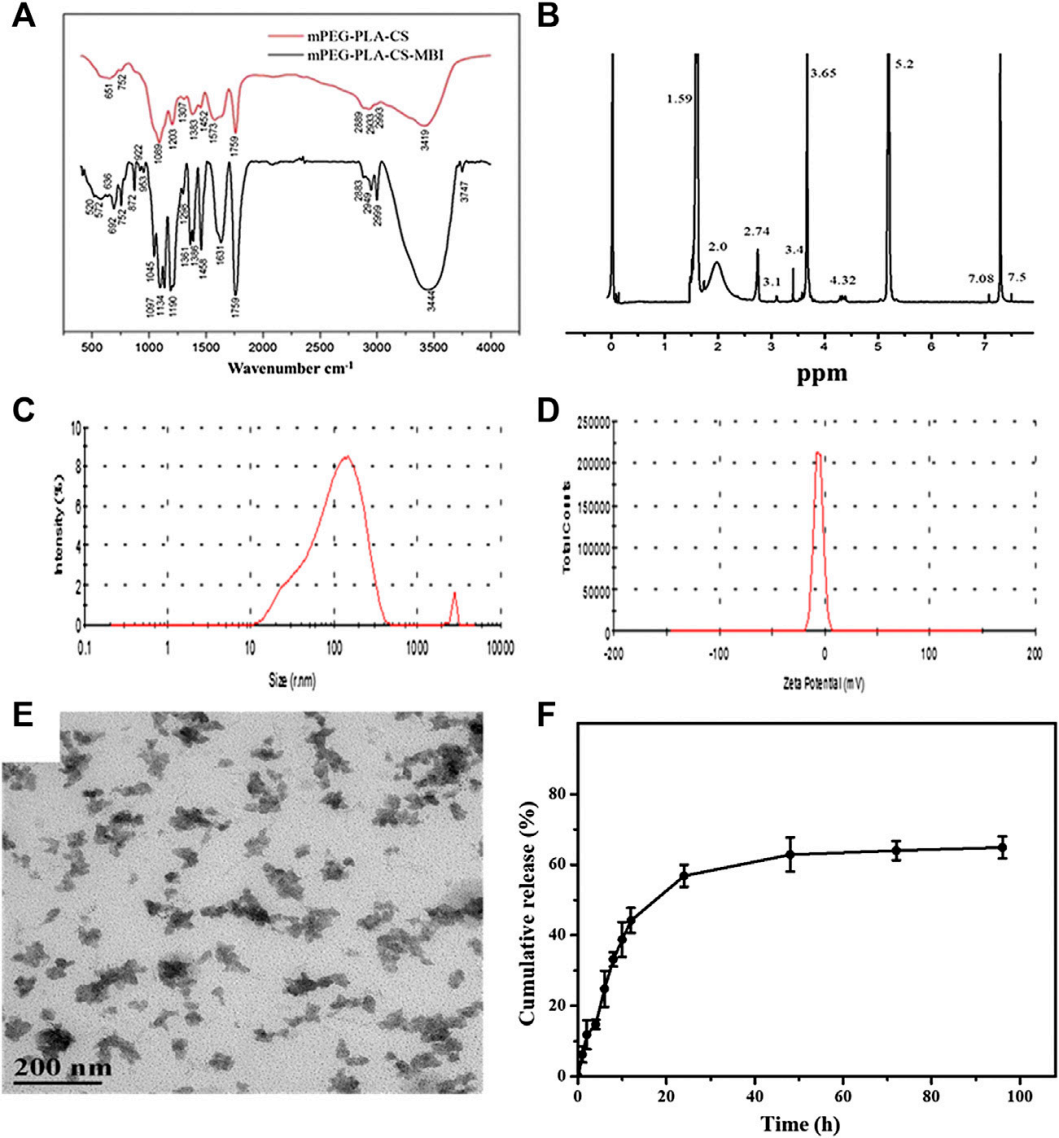
Characterization of emodin-NP **(A)** FTIR spectra showing amide bands I and II of the copolymer at 1757 and 3443 cm^−1^, respectively **(B)** ^1^H NMR spectrum with characteristic proton peaks of PEG and PLA at 3.65 and 5.2 ppm, respectively **(C)** size distribution spectrum **(D)** zeta potential of mPEG-PLA-CS-MBI determined by laser diffraction **(E)** morphology of mPEG-PLA-CS-MBI nanoparticles determined by TEM **(F)** *in vitro* release of MBI from mPEG-PLA-CS-MBI in pH 7.2 PBS at 37°C.

The synthesis process of mPEG-PLA-CS-MBI copolymer is shown in Supplementary Figure S1. The structure of the mPEG-PLA-CS-MBI chemical conjugate was confirmed by infrared and 1H NMR spectroscopy. The infrared peaks corresponding to amide bands I and II of the copolymer appeared at 1757 and 3443 cm-1, respectively (Figure 1A). The ^1^H NMR spectrum showed characteristic proton peaks of PEG and PLA at 3.65 and 5.2 ppm, respectively (Figure 1B).

The average particle sizes of emodin-NP were between 100 and 200 nm, and the polydispersity index (PDI <0.25) was acceptable (Figure 1C). The zeta potential was −12.83 ± 0.11 mV (Figure 1D). TEM images showed that emodin-NP had uniform cotton-like shapes, and the average diameter was consistent with the DLS measurement results. Emodin-NP was composed of many small nanoparticles (Figure 1E). *In vitro* release data showed that emodin-NP displayed a release pattern in deionized water of ∼65% emodin (Supplementary Figure S2) during 96 h of incubation (Figure 1F). In addition, the mPEG-PLA-CS-MBI nanoparticles did not affect the cell viability *in vitro* (Supplementary Figure S3).

### Emodin-NP Colonic Irrigation Improved Renal Function and Inhibited Tubulointerstitial Injury

**FIGURE 2:**
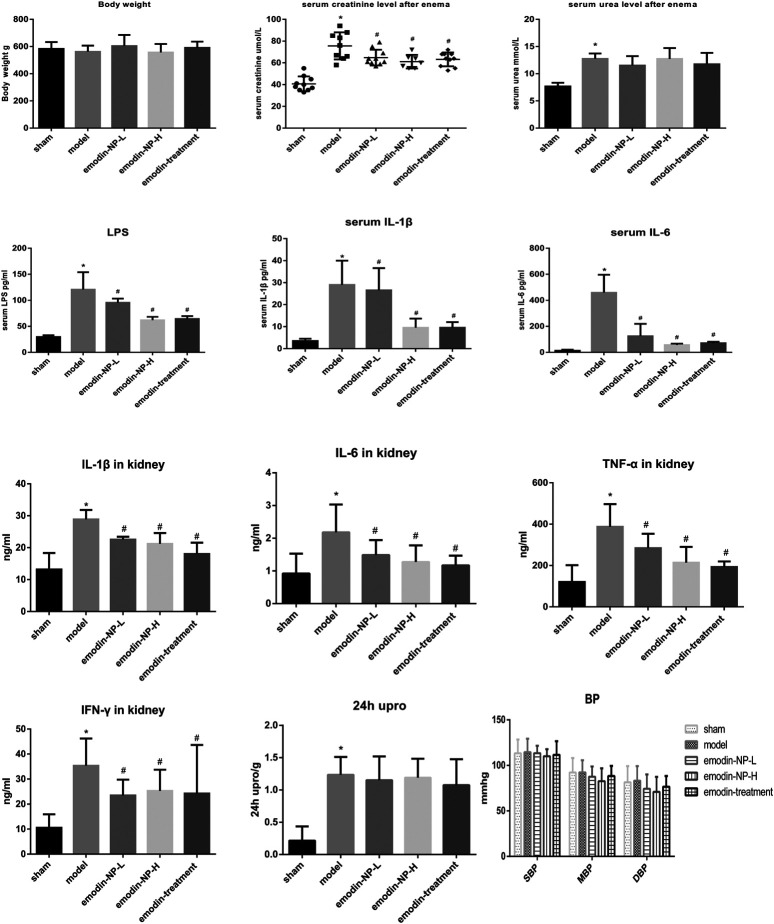
Serum creatinine, 24 h Urine protein, urea, blood pressure, systemic and renal inflammation between groups. The level of serum creatinine in emodin-NP and emodin-treated rats notably decreased. ^*^ *p* < 0.05 vs. normal group. ^#^ *p* < 0.05 vs. model group.

**FIGURE 3:**
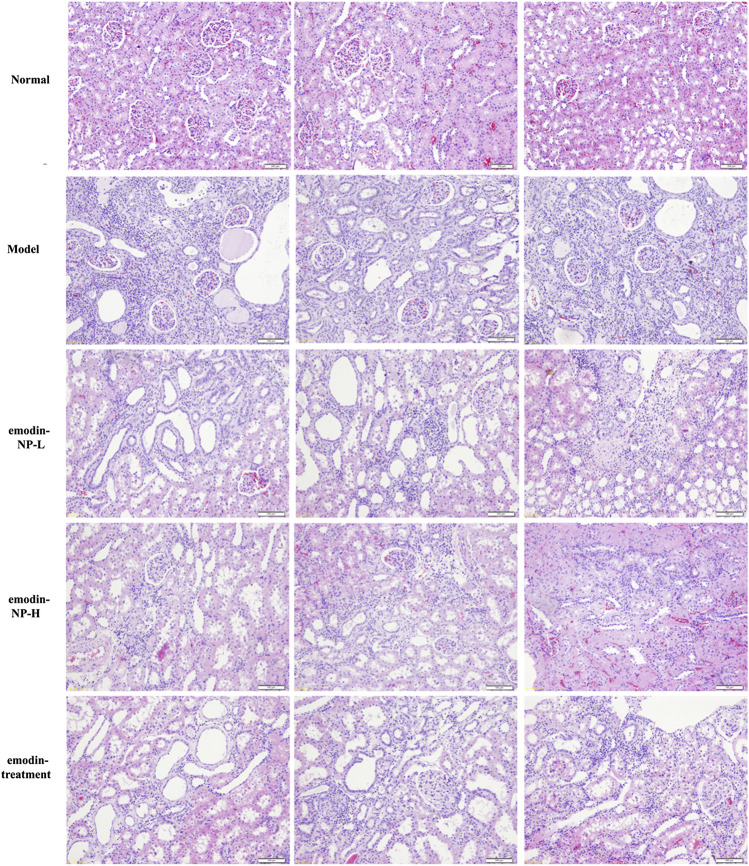
Renal tissue HE/Masson staining. Renal histopathological changes in sham rats and model rats treated with or without emodin-NP/emodin (hematoxylin and eosin, ×100, Masson stain, ×200).

**FIGURE 4:**
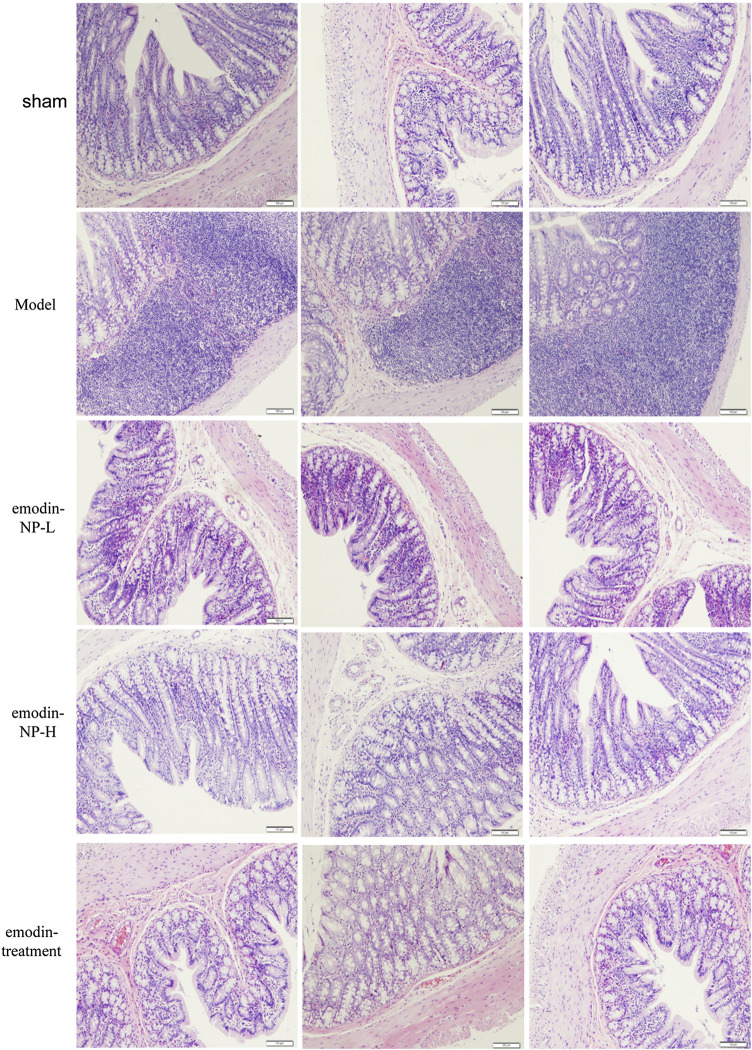
Expressions of TLR4, MyD88, and NF-κB in colon tissues, collagen Ⅰ in renal tissues **(A)** Western blot analysis of TLR4, MyD88, and NF-κB in colon tissues **(B)** Relative activity of protein expression. Data were expressed vs. *β*-actin and compared by analysis of variance **(C)** Western blot analysis of collagen Ⅰ in renal tissues. Relative activity of protein expression. Data were expressed vs. *β*-actin and compared by analysis of variance. ^*^ *p* < 0.05 vs. normal group. ^#^ *p* < 0.05 vs. model group.

The colonic irrigation data are summarized in Figures 2, 3 and Figure 4. Compared with the normal group, the CKD model, emodin-NP, and emodin groups showed a significant increase in serum creatinine concentrations, serum urea and 24 h urine protein. Renal tissue HE and Masson staining showed that the renal tubular lumen was enlarged, as well as mononuclear lymphocyte infiltration, renal tubular atrophy, and interstitial fibrosis. Compared with the CKD model group, low-dose and high-dose emodin-NP colonic irrigation significantly lowered the creatinine concentrations, and the histopathological changes were improved. The CKD groups with emodin-NP, emodin, and control colonic irrigation had no difference in body weight, urea, 24 h urine protein, as well as blood pressure (SBP, systolic blood pressure, DBP, diastolic pressure, MBP, mean arterial pressure), compared with the sham group (Figure 2). The collagen Ⅰ expression in renal tissues was significantly lower in the sham group than in the 5/6 Nx model group (*p* < 0.05), while significantly higher in the 5/6 Nx model group than in the 5/6 Nx + emodin-NP or emodin group (*p* < 0.05; Figure 4). The therapeutic effect of high-dose emodin-NP after two days was slightly better than that of emodin treatment every day.

### Emodin-NP Colonic Irrigation Significantly Reduced Systemic Inflammation

After 8 weeks of treatment, IL-1β, IL-6, TNF-α, IFN-γ, and LPS in the serum or renal tissues samples were analyzed by ELISA, as shown in Figure 2. Compared with the normal group, the model groups with emodin-NP, emodin, and control colonic irrigation exhibited a significant increase in IL-1β, IL-6, TNF-α, IFN-γ, and LPS levels. Compared with the CKD model group and emodin group, emodin-NP colonic irrigation significantly reduced the IL-1β, IL-6, TNF-α, IFN-γ, and LPS levels.

### Emodin-NP Colonic Irrigation Improved Inflammation and Intestinal Barrier

**FIGURE 5:**
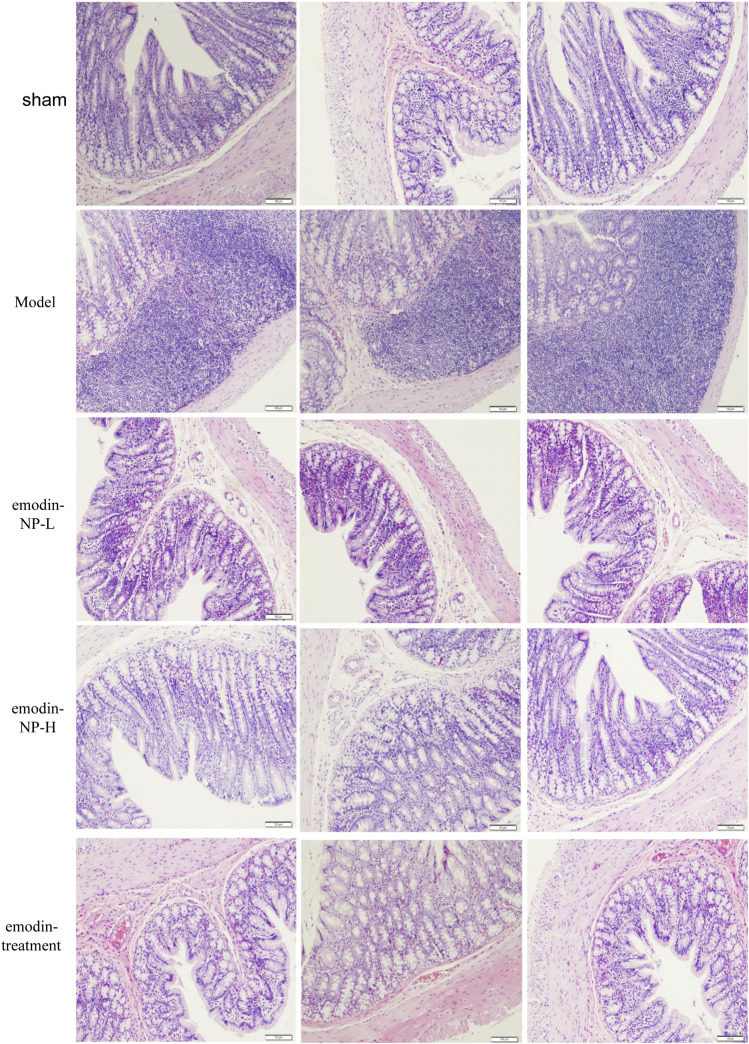
Colon renal tissue HE staining. Colon histopathological changes in normal rats and model rats treated with or without emodin-NP/emodin (hematoxylin and eosin stain, ×100).

In the model group, colon tissue HE staining showed obvious edema in the mucosal layer of the colon and lamina propria compared with the normal group. Moreover, infiltration of lymphocyte monocytes was observed in the mucosal layer of the 5/6 Nx model group. After colonic irrigation with either emodin-NP or emodin, the infiltration of inflammatory cells in the mucosal layer was reduced, and the edema in the mucosal layer and lamina propria was significantly improved (Figure 5).

The expression of key indicators of intestinal inflammation (TLR4, MyD88, and NF-κB) in the colon tissues was significantly lower in the sham group than in the 5/6 Nx model group (*p* < 0.05). This expression was significantly higher in the 5/6 Nx model group than in the 5/6 Nx + emodin-NP or emodin group (*p* < 0.05, Figure 4).

### Changes in Gut Microbiota After Enema Treatment

**FIGURE 6:**
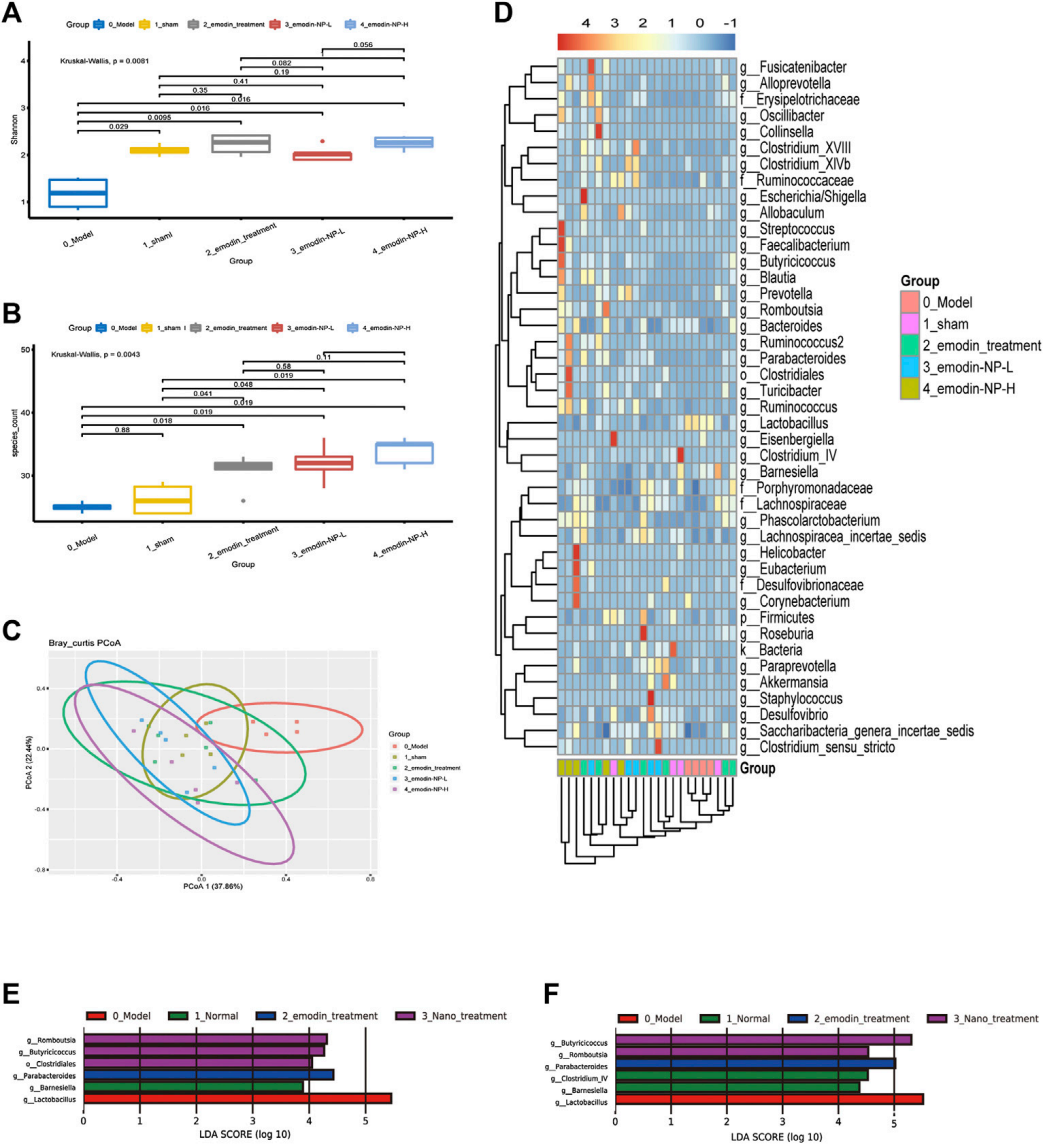
Diversity of gut microbiota **(A)** Shannon index and **(B)** species count of the five groups **(C)** PCoA based on Bray–Curtis distance of the genus levels for 16S rDNA gene sequencing **(D)** Heatmap of key bacterial genera responding to the emodin-NP/emodin treatment. Gut microbiota difference analysis at the **(E)** genus level and **(F)** multiple levels between the five groups.

The Shannon index was the highest in the emodin group and lowest in the model group at the genus level; the Shannon index of the normal group and emodin-NP treatment groups was intermediate. The index and species count differed significantly between the five groups (Kruskal–Wallis test). The PCoA results showed differences in intestinal microbial diversity between the five groups; the heatmap based on cluster analysis showed that different groups of samples were clustered together, and intestinal microbial diversity differed between the groups (Figure 6).

**FIGURE 7:**
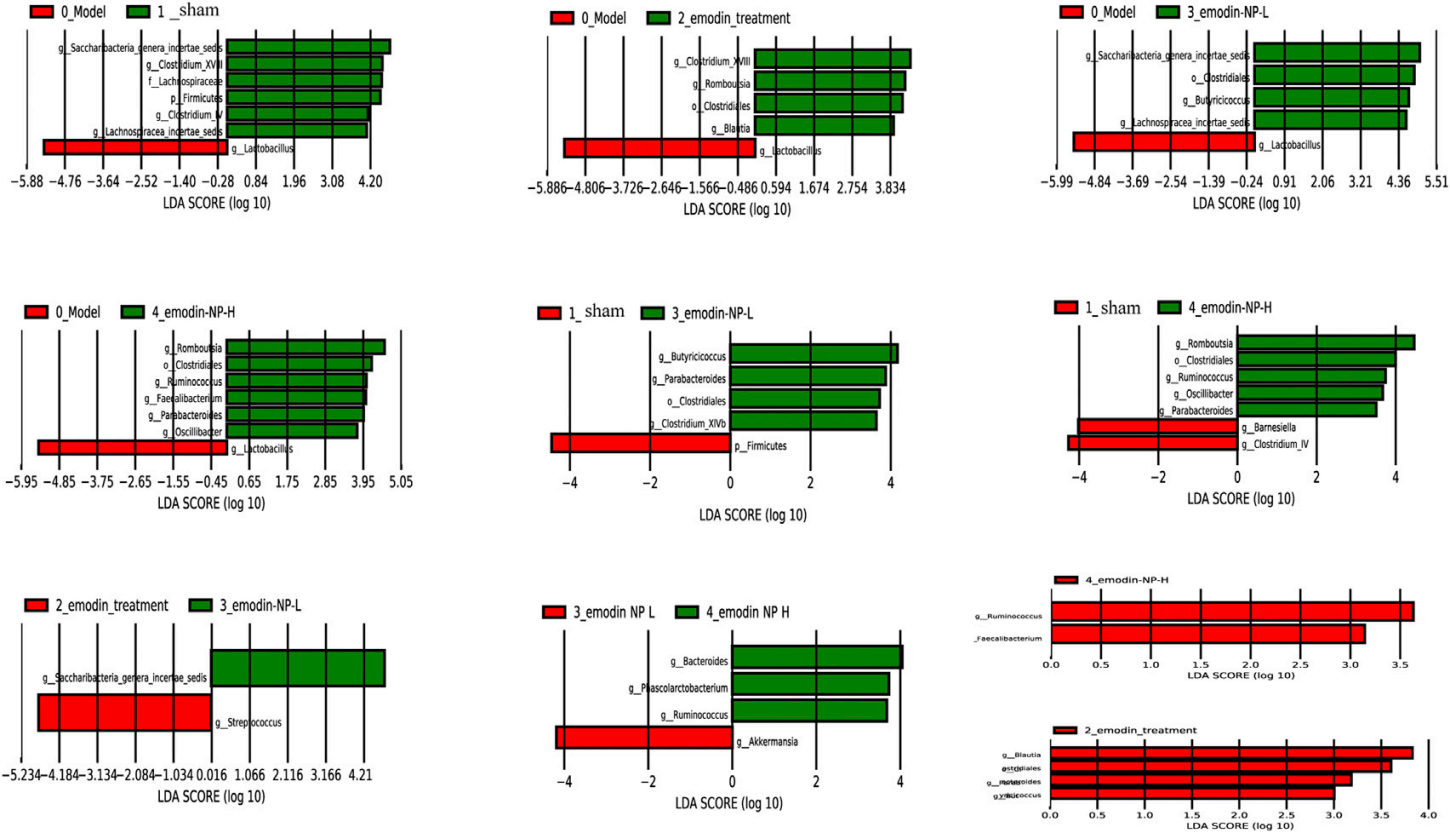
Pairwise comparison difference analysis of gut microbiota between groups. No special bacteria were screened in the normal group compared with the emodin treatment group, and no special bacteria were screened in the emodin treatment group compared with the emodin-NP-H group.

LEfSe was further used to analyze the differences between the five groups, with LDA scores >2 regarded as reflecting significant differences. Genus-level results of the differences analysis between groups showed that the bacteria enriched in the sham group were *Clostridium* and *Barnesiella*. *Butyricicoccus* and *Romboutsia* were enriched in the emodin-NP treatment groups (low-dose and high-dose), while *Parabacteroides* and *Anaerostipes* were enriched in the emodin group. Multilevel multi-group difference analysis showed that *Barnesiella* was enriched in the normal group. The bacteria enriched in the emodin-NP treatment groups were *Romboutsia*, *Butyricicoccus*, *Parabacteroides*, and *Clostridium*. Pairwise comparisons showed that compared with the normal group, *Saccharibacteria, Clostridium,* Lachnospiraceae, and *Firmicutes* were reduced in the model group. *Clostridium, Romboutsia*, and *Blautia* were higher in the emodin treatment group than in the model group. Emodin-NP treatment increased *Saccharibacteria, Clostridiales, Butyricicoccus,* and Lachnospiraceae in the low-dose group and *Clostridium, Aloprevotella, Romboutsia, Oscillibacter, Ruminococcus,* and *Turicibacte*r in the high-dose group. The difference between the emodin-NP and emodin treatments was that *Saccharibacteri*a was increased in the emodin-NP treatment group, while *Streptococcus* increased in the emodin treatment group. The difference between the emodin-NP low-dose and high-dose treatment was that *Bacteroides*, *Phascolarctos* bacterium*,* and *Ruminococcus* were increased in the emodin-NP low-dose treatment group, while *Akkermansia* increased in the emodin-NP high-dose treatment group (Figure 7).

**FIGURE 8:**
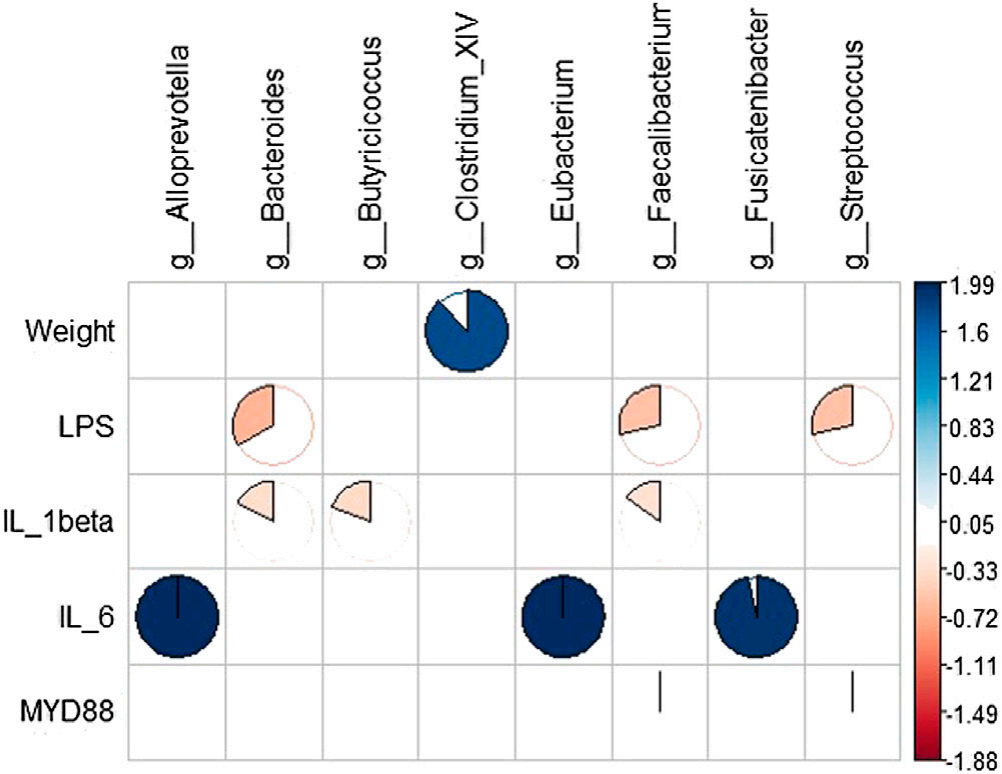
Correlation analysis between gut microbiota and systemic inflammation. Blue represents a positive correlation between gut microbiota and systemic inflammation, and yellow represents a negative correlation.

RDA results showed that intestinal microbes were significantly correlated with systemic inflammation and LPS in the serum samples, and body weight was positively correlated with *Clostridium*. IL-1β was positively correlated with *Bacteroides, Butyricicoccus*, and *Faecalibacter*; IL-6 was negatively correlated with *Alloprevotella, Eubacterium*, and *Fusicatenibacter*; and serum LPS was negatively correlated with *Bacteroides, Faecalibacter*, and *Streptococcus* (Figure 8).

## Discussion

In the current study, we intended to investigate the mechanisms of emodin-NP colonic irrigation attenuates renal injury by focusing on the change of intestinal flora and systemic inflammation. We found that emodin-NP colonic irrigation lowered the levels of LPS, IL-1β, and IL-6 markedly, and modulated the gut microbiota.

In recent years, more and more studies have indicated that in CKD patients, the accumulation of uremic toxins and systemic inflammation is related to the bacterial load and endotoxins produced by gastrointestinal dysfunction, which increases the permeability of the intestine and leads to the development of CKD (Sabatino et al., 2015; Chen et al., 2019). Therefore, the gut-kidney axis, an intimate connection between the gut and kidney has been proposed (Meijers and Evenepoel, 2011).

The gut microbiome is also known as the “second human genome”, and it plays an important role in maintaining health and disease pathogenesis. The proliferation of undesirable biological bacteria in CKD patients interferes with the symbiotic relationship between the host and the microbiome. The fermentation of proteins and amino acids by intestinal bacteria produces excessive amounts of potentially toxic compounds, such as ammonia, phenols and indole, but reduces the production of short-chain fatty acids. The impaired intestinal barrier function of patients with CKD will cause the transfer of intestinal uremic toxins to the systemic circulation, resulting in CKD, cardiovascular disease and protein energy waste (Ramezani et al., 2016).

Among the potential applications of the human gut microbiome, it has been proposed to use it as a disease biomarker and to change the composition of the microbiome to treat diseases (Zou et al., 2013). The microbiome is a therapeutic target for the management of patients with CKD. In CM theory, eliminating toxins is the key treatment for CKD (Guo and Rao, 2018). Chinese herbs enemas as a colon-targeted therapy have been used to cure CKD for half a century. Its representative method is to use the rhubarb-based compound enema through the intestine. In the past half century, numerous clinical studies have demonstrated that rhubarb-based decoction colonic irrigation treatment not only ameliorates uremia symptoms but also reduces uremic toxins in blood (Kavianinia et al., 2016).

Our previous studies have demonstrated that colonic irrigation with emodin, the most important active ingredient of rhubarb, modulates the multiple goals of intestinal microbiota and its metabolic toxins through the gut-kidney axis to improve the renal function (Zeng et al., 2016). However, the poor solubility, limited colonic irrigation time, and inadequate colon adhesion of emodin hinder its effects on the intestinal microecology.

Chitosan, a polymer of 2-amino-2-deoxy-β-d-glucose, is the most widely used compound in colonic adhesion nanomaterials. Chitosan has excellent biocompatibility, can form hydrogen bonds and electrostatic interactions with intestinal mucosal proteins, and has good bioadhesive properties (Bernkop-Schnürch, 2005; Iglesias et al., 2019). However, this non-covalent bond-based adhesion does not guarantee the sustained release of drugs at specified sites. The application of chitosan is limited, whereas after its thiolation, the adhesion performance is significantly improved because the vulcanized polymer can form disulfide bonds with the mucosa layer and cysteine-rich subregions in mucin, allowing for specific binding (Hauptstein et al., 2014a).

The application of thiolated polymers as drug carriers is not ideal because they have weak interactions with hydrophobic drug molecules such as emodin, which often results in fast drug release and poor encapsulation efficiency. To solve the problems of the low water solubility of chitosan and the weak effect of thiolated polymers and poorly soluble drugs, we introduced hydrophilic mPEG and PLA to form an mPEG-PLA-CS polymer. Based on this, the polymer was thiolated by MBI to form a mercapto polymer (mPEG-PLA-CS-MBI). The thiol polymer forms disulfide bonds and adheres to the mucosal surface through thiol oxidation, thereby extending the residence time of the drug on the mucosa, affording the prepared particles with a surface stabilizing effect, and resulting in nanoparticles with stronger adhesion properties (Rahmat et al., 2011; Hauptstein et al., 2014b; Kongsong et al., 2014; Jiang et al., 2016).

Finally, we developed a nanoparticle-mediated drug delivery system using a novel conjugate of a diblock copolymer with a core-shell structure, mPEG-PLA-CS-MBI nanoparticles. We found that the nanoparticle-mediated targeting of emodin (emodin-NP) has a higher stability, better colon adhesion, and more persistent effects than emodin alone. Emodin-NP has good intestinal adhesion up to 48 h via colonic irrigation.

We investigated the mechanisms by which emodin-NP colonic irrigation attenuates renal injury by focusing on the changes in intestinal microbiota and systemic inflammation (Ramezani and Raj, 2014; Guo and Rao, 2018). We found that treatment with emodin-NP improved the kidney function and limited the expansion of tubulointerstitial fibrosis. Treatment with emodin-NP once every two days is comparable to emodin treatment once a day. Furthermore, emodin-NP via colonic irrigation markedly reduced the serum IL-6, IL-1β, and LPS levels and improved the intestinal barrier function.

Our research results indicated that the flora microbiota significantly changed in the CKD groups. Emodin-NP treatment significantly regulated the intestinal microbiota of CKD patients. CKD is often accompanied by changes in intestinal flora (Feng et al., 2019). According to a previous research, renal failure is always accompanied by a decrease in flora microbiota diversity (Bor et al., 2019), which is consistent with our results.

Our study results showed that *Saccharibacteria*, *Clostridium*, Lachnospiraceae*,* and *Firmicutes* were reduced in the CKD rats. The progression of CKD may be due to the reduction of these probiotics and their products. Recent studies have indicated that *Saccharibacteria* may be beneficial to host bacteria by promoting biofilm formation, which evades the immune system and reduces inflammation (Meehan and Beiko, 2014; Bedree et al., 2018). Lachnospiraceae is a family of Clostridia, which contains the main component of mammalian gastrointestinal microbiota. Some members of it can produce butyrate (Sakamoto et al., 2009). *Barnesiella* is a member of *Porphyromonadaceae* within the phylum Bacteroidetes (Wylie et al., 2012). A recent study showed that the most abundant novel classification in healthy humans are *Barnesiella* (Weiss et al., 2014) and it might can reduce systematic inflammatory in mice (Boesmans et al., 2018).

According to our findings, emodin-NP treatment could increase probiotics, especially butyrate-producing bacteria. The *Clostridium* cluster IV genus *Butyricicoccus* is a Gram-positive, which can produce high levels of butyrate. Butyrate has been shown to reduce intestinal inflammation in inflammatory bowel disease (IBD) patients (Eeckhaut et al., 2013). *Butyricicoccus* bacteria were reduced in patients with IBD. The administration of *B. pullicaecorum* can alleviate TNBS-induced colitis in rats, and the supernatant of the culture of it can enhance the epithelial barrier function (Milani et al., 2016). *Romboutsia* is a recently described bacterial genus which commonly identified in the human intestine and often connected with a healthy status (Ricaboni et al., 2016; Wang et al., 2019). *Romboutsia* reduction in mucosa were associated with polyps which may be a potential microbial indicator. *Parabacteria* have reduced weight gain, hyperglycemia and hepatic steatosis in ob/ob and high-fat diet mice. In addition, *Parabacteroides* greatly changed the distribution of bile acids, increasing gallstone acid and ursine. The content of oxycholic acid, and increased the content of succinic acid in the intestines of mice on a high-fat diet. (Chelakkot et al., 2018). *Akkermansia* is an anaerobic probiotic that stably colonizes the intestinal mucus layer and is associated with varieties of diseases, such as obesity, diabetes, and IBD. One study showed that rhubarb extract can increase *Akkermansia* in mice with acute alcoholism and reduce liver inflammation damage and oxidative stress (Chelakkot et al., 2018). The study also found that *Akkermansia* outer vesicles can increase intestinal barrier integrity and metabolic function in diabetic mice. Supplementation with *Akkermansia* can also improve insulin levels, insulin resistance, and total cholesterol levels in obese patients (Depommier et al., 2019). Ottman et al. (2017) also found that *Akkermansia* can activate TLR2 and TLR4 to increase the production of IL-10, thereby regulating the immune response and intestinal barrier function. Therefore, *Akkermansia* affect host health by preventing inflammatory damage caused by the translocation of bacteria and its products derived from the intestinal tract.

16S rDNA analyses revealed that emodin-NP returned the microbial balance of CKD. Together, these results showed that emodin-NP attenuated adverse kidney tubulointerstitial fibrosis and dysfunction by the modification of gut microbiota disorders. Emodin-NP is a novel approach to treating CKD.

## Conclusion

Emodin colonic irrigation modulates gut microbiota and delays CKD progression. However, the poor solubility and limited colonic irrigation time of emodin hinder its clinical application. In order to improve it, we prepared monomethoxy-poly (ethylene glycol)-poly (lactic acid)-chitosan-2-mercaptobenzimidazole nanoparticles with incorporated emodin (emodin-NP) and studied their efficacy in delaying CKD progression. Our results demonstrate that emodin-NP colonic irrigation attenuates kidney tubulointerstitial fibrosis and dysfunction by modifying gut microbiota disorders and inflammation. Treatment with emodin-NP once every two days is comparable to emodin treatment once a day. Emodin-NP is a novel approach to treating CKD.

## Data Availability Statement

The datasets presented in this study can be found in online repositories. The names of the repository/repositories and accession number(s) can be found below: https://www.ncbi.nlm.nih.gov/, 593972

## Ethics Statement

This study was approved by the institutional ethics review board of Second Affiliated Hospital of Guangzhou University of Chinese Medicine.

## Author Contributions

The conception and design were proposed by CZ and GR. Animal and molecular biology experiments were mainly performed by ZL and CJ. 16S rDNA sequencing and data analysis were performed by YC and LH. mPEG-PLA-CS-MBI were designed and measured by XLu and YL. The manuscript was drafted by ZL and CJ, and reviewed by CZ. All authors read and approved the final manuscript.

## Funding

This study was supported by the National Natural Science Foundation of China (81873142, secured by CZ) and the Natural Science Foundation of Guangdong Province (2019A1515011054, secured by CZ).

## Conflict of Interest

The authors declare that the research was conducted in the absence of any commercial or financial relationships that could be construed as a potential conflict of interest.
