# Supplementary figures and images for: Nanoparticle-Mediated Delivery of Emodin via Colonic Irrigation Attenuates Renal Injury in 5/6 Nephrectomized Rats
Source: Front Pharmacol. 2021 Jan 21;11:606227. doi: 10.3389/fphar.2020.606227 (PMC7858270; doi:10.3389/fphar.2020.606227)

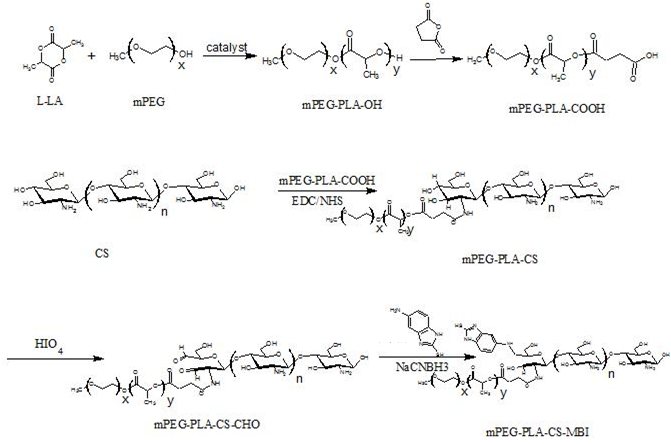

Supplement: Supplementary file 1 [file image1.tif]

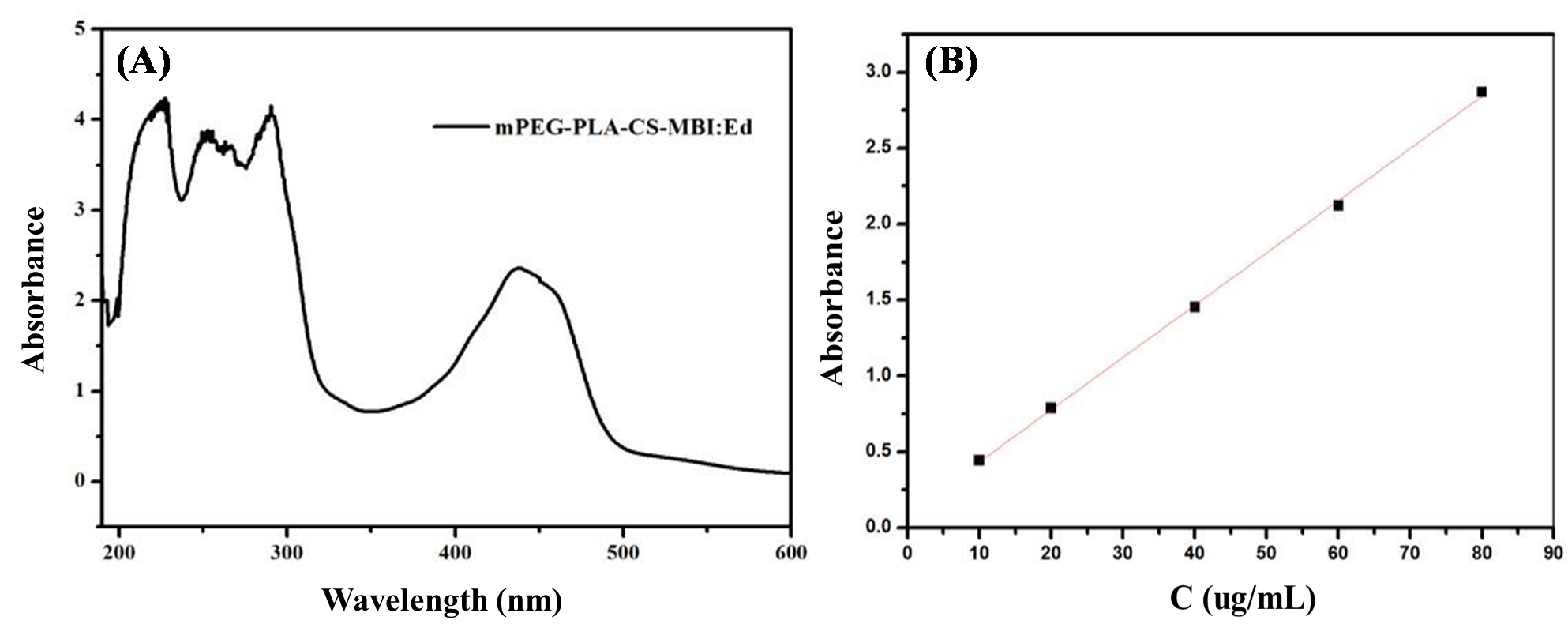

Supplement: Supplementary file 2 [file image2.tif]

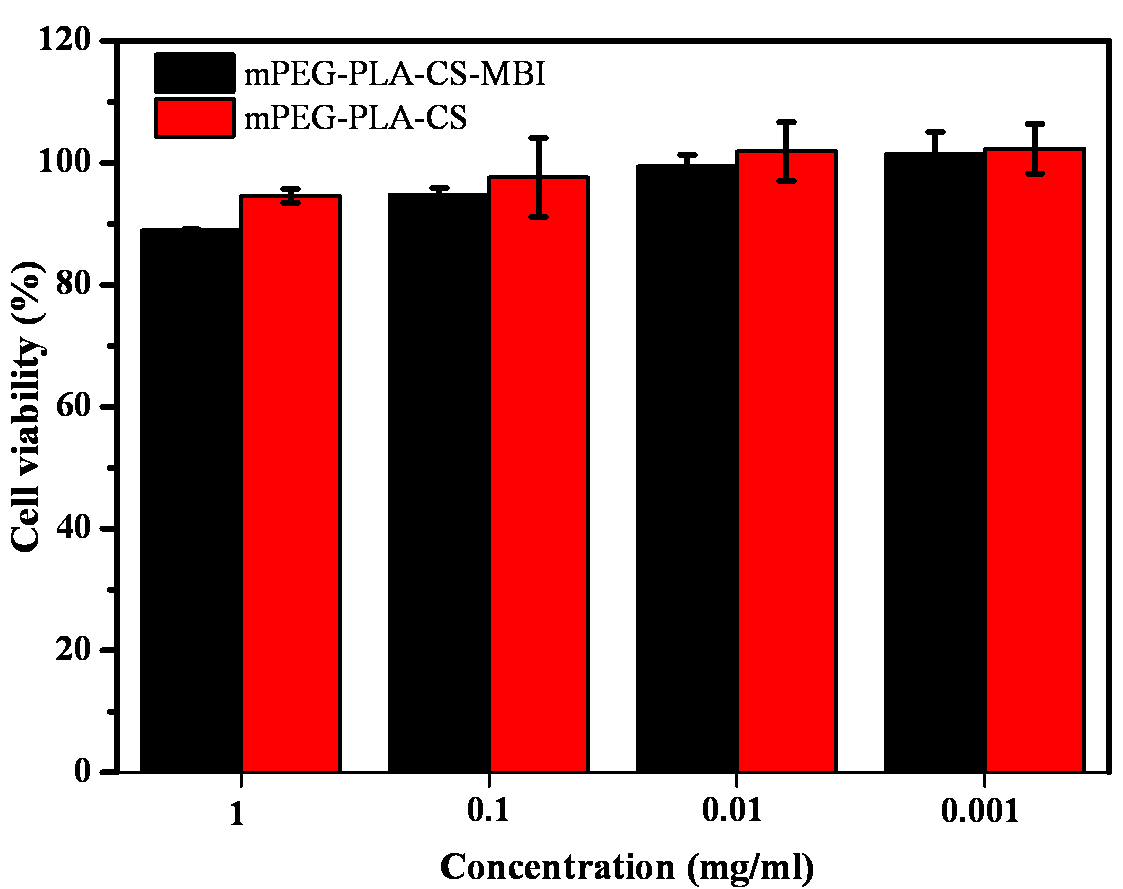

Supplement: Supplementary file 3 [file image3.tif]
